# Epicardial connection between superior vena cava and right atrium contributes to subsequent atrial fibrillation: a case report

**DOI:** 10.1093/ehjcr/ytaf016

**Published:** 2025-01-16

**Authors:** Shunya Otsubo, Masao Takemoto, Eiji Nyuta, Takuya Tsuchihashi

**Affiliations:** Cardiovascular Centre, Social Medical Corporation Steel Memorial Yawata Hospital, 1-1-1 Haruno-machi, Yahatahigashi-ku, Kitakyushu 805-8508, Japan; Cardiovascular Centre, Social Medical Corporation Steel Memorial Yawata Hospital, 1-1-1 Haruno-machi, Yahatahigashi-ku, Kitakyushu 805-8508, Japan; Cardiovascular Centre, Social Medical Corporation Steel Memorial Yawata Hospital, 1-1-1 Haruno-machi, Yahatahigashi-ku, Kitakyushu 805-8508, Japan; Cardiovascular Centre, Social Medical Corporation Steel Memorial Yawata Hospital, 1-1-1 Haruno-machi, Yahatahigashi-ku, Kitakyushu 805-8508, Japan

**Keywords:** Ablation, Atrial fibrillation, Case report, Epicardial connection, Superior vena cava

## Abstract

**Background:**

The superior vena cava (SVC) acts as a non-pulmonary vein (PV) trigger for atrial fibrillation (AF) in 2%–6% of patients and harbours 25%–40% of non-PV foci. Approximately 10% of patients with AF have epicardial connections (ECs) between the atrium and PV inside the PV isolation lines, which are associated with AF recurrence. However, the contribution of EC(s) between the SVC and right atrium (RA) to subsequent AF remains unknown.

**Case summary:**

A 76-year-old woman underwent ablation for recurrent AF. She had undergone cryo-balloon ablation for paroxysmal AF 3 years previously. After confirming the complete entrance and exit blocks of the four PVs, SVC firing-induced AF was observed. After SVC isolation, the EC between the SVC and RA was observed. No AF was induced after EC ablation.

**Discussion:**

Although the mechanisms of ECs in the SVC and RA have not been entirely elucidated, several potential mechanisms have been proposed. (i) Anatomically inherited myofibres/bundles may run through the epicardial side between the SVC and RA. (ii) Epicardial connections between the right PV and the SVC or RA have been recently reported. Thus, we might speculate on the possibility of the existence of EC(s) between the right PV and both the SVC and RA. After cryoablation in the first session, the connection between the SVC and RA remained, which might have acted as EC(s). Thus, physicians should consider the possibility of EC(s) when remaining potentials in the SVC are observed, even though the SVC isolation line seems to be completed.

**Learning points**

- An epicardial myofibre/bundle, such as the septopulmonary bundle, Marshal bundle, Bachmann bundle, or intercaval bundle, connects the pulmonary veins and atrium on the epicardial side.
- Physicians should consider the possibility of epicardial connections when remaining potentials in the superior vena cava (SVC) are observed, even though the SVC isolation line seems to be completed.

## Introduction

Pulmonary vein (PV) antrum isolation (PVAI) is a useful treatment strategy for atrial fibrillation (AF) worldwide.^1,2^ However, in ∼10%–30% of AF cases, non-PV triggers during/after PVAI using cryo-balloon ablation (CBA) are observed and contribute to AF recurrence.^3,4^

## Summary figure

**Figure:**
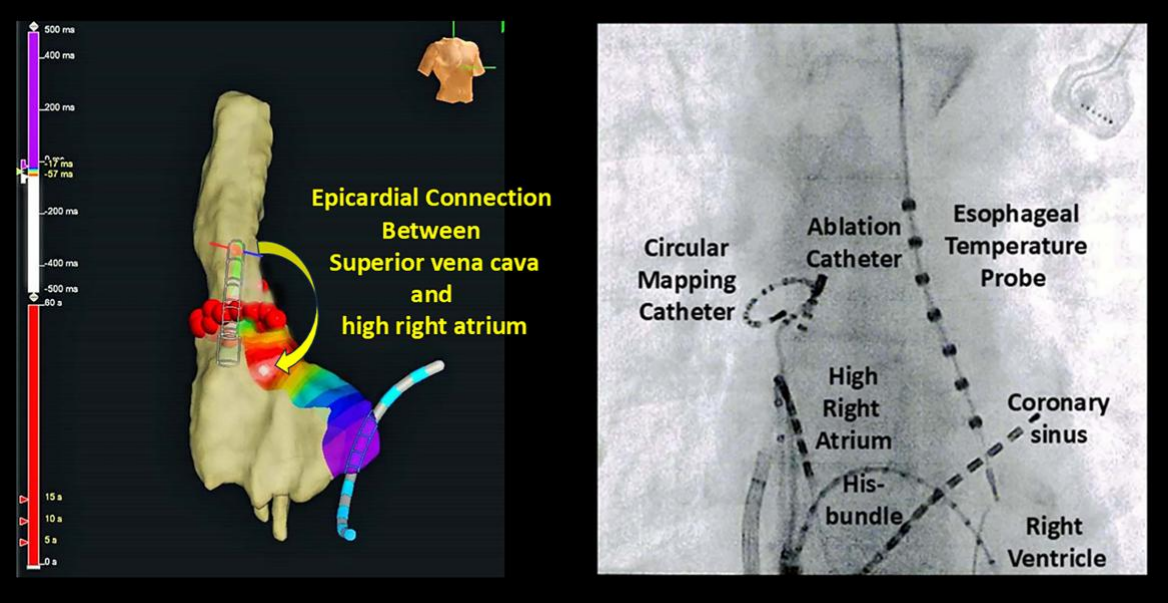


## Case presentation

**Figure 1:**
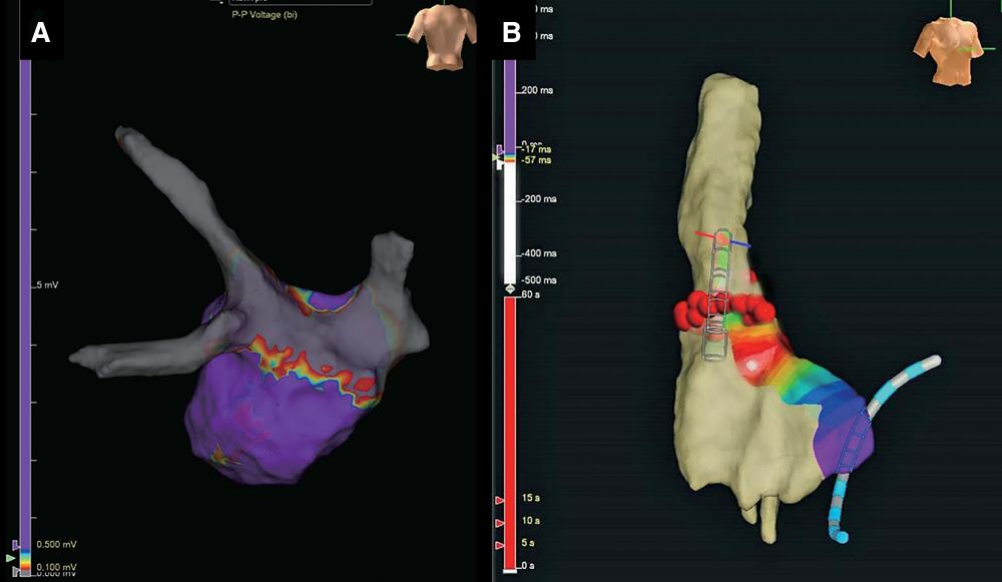
Voltage map viewed from the posteroanterior view (*A*) and local activation time isochronal mapping of the right atrium and superior vena cava constructed by EnSite™ viewed from the right anterior oblique view (*B*).

**Figure 2:**
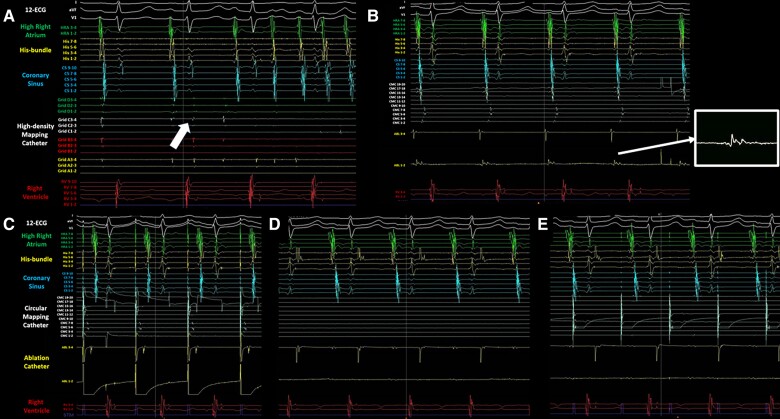
Intra-cardiac electrograms of the spontaneous activities from a high-density mapping catheter placed in the superior vena cava that initiated atrial fibrillation (*A*), the earlier fragmentated potential with fragmentated prepotential recorded from the tip of the ablation catheter than that from the circular mapping catheter (*B*), the earliest activation site of the right atrium during pacing by an ablation catheter on this site (*C*), the abolishment of the remaining potentials inside the superior vena cava isolation line (complete entrance block) (*D*), and the non-conduction to the atrium pacing from the circular mapping catheter inside the superior vena cava isolation line (complete exit block) (*E*). The white square picture in (*B*) indicates the enlarged potential recorded from the tip of the ablation catheter.

**Figure 3:**
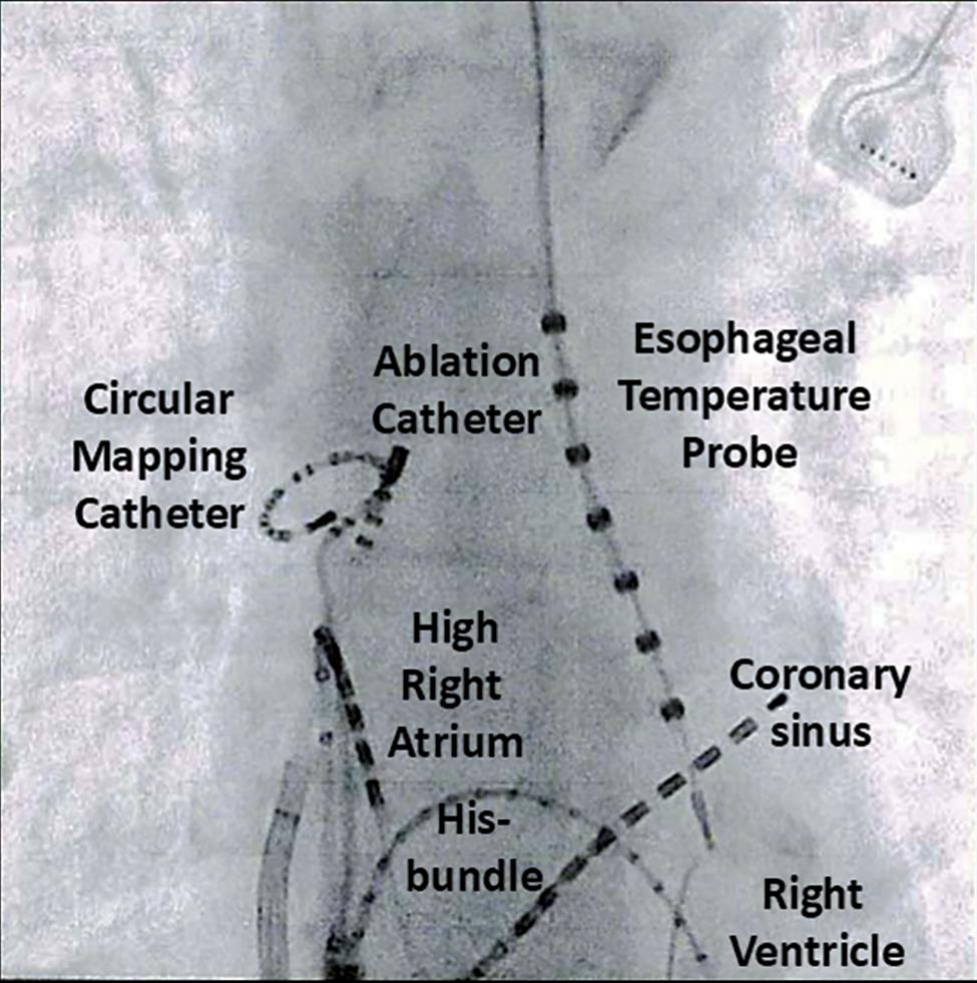
Frontal fluoroscopic images during delivering of radiofrequency energy to epicardial connection.

A 76-year-old woman was referred to our hospital with a few-month history of palpitations due to recurrent paroxysmal AF (PAF), on a background of cerebral infarction and hyperlipidaemia. She had undergone CBA for PAF 3 years prior and has had no symptoms or arrhythmias for 3 years. She was taking bisoprolol (2.5 mg), pitavastatin (2 mg) once daily, and apixaban (5 mg) two times daily. She was readmitted to our hospital for ablation as her AF was drug refractory. On admission, she had blood pressure of 116/68 mmHg and regular heart rate of 62 b.p.m. Precordial auscultation revealed normal cardiac and respiratory signs. Her low- and high-density lipoprotein cholesterol levels were 86 and 52 mg/dL, respectively. A 12-lead electrocardiogram revealed sinus rhythm. Echocardiography revealed a normal left ventricular ejection fraction and left atrial (LA) diameter (30.1 mm). The patient’s CHADS_2_/CHA_2_DS_2_-VASc score was 3/5. Preprocedural contrast-enhanced computed tomography (CT) revealed right inferior PV stenosis. After a double transseptal puncture, the LA and PVs were reconstructed in detail by an EnSite™ (Abbott) using a high-density mapping catheter (HDMC) (Advisor™ HD Grid Catheter, Abbott) during sinus rhythm. We confirmed the absence of any remaining potential inside the PVAI lines (complete entrance block) and the exit block of the four PVs by pacing using an HDMC with an output of 10 V (complete exit block). In addition, we confirmed the absence of epicardial connections (ECs)^5^ inside the PVAI lines. We then empirically added LA posterior wall isolation with roof and bottom lines (*Figure 1A*). Next, according to our routine approach,^6^ the voltage and activation maps of the superior vena cava (SVC) and right atrium (RA) were precisely reconstructed by an EnSite™ system using a HDMC during sinus rhythm. The length from the top of the sinus node to the top of the myocardial sleeve and the diameter in the SVC were 41 and 18 mm, respectively. The bolus injections of isoproterenol (3 μg) induced AF, following spontaneous activities from an HDMC positioned in the SVC (white arrow in *Figure 2A*). Thus, circumferential SVC isolation was additionally performed at 45°C with a power limit of 25 W. To prevent diaphragmatic paralysis, we verified that the right diaphragm did not twitch upon pacing from the ablation catheter at 10 V just before radiofrequency energy application. However, the remaining potentials of the circular mapping catheter (CMC) (Optima™, Abbott) positioned in the SVC were observed (*Figure 2B*). Although the SVC isolation line was mapped precisely using CMC, the line seemed complete. Because we considered the possibility of an EC between the SVC and RA, mapping using an ablation catheter and CMC was performed. We then confirmed the earlier potential with a fragmented prepotential of the ablation catheter compared with that of the CMC (*Figure 2B*). The distance between the tip of the ablation catheter and the SVC isolation line was 18 mm. Local activation time isochronal mapping was performed using a CMC during pacing by an ablation catheter at this site and demonstrated that the earliest activation site was the RA (*Figure 2C*) without electrical endocardial conduction, which spread in a centrifugal pattern from this breakout site (*Figure 1B*), leading to a diagnosis of EC between the SVC and RA. Radiofrequency energy was delivered to EC at a maximum power of 25 W (*Figure 3*). The remaining potentials in the SVC steadily abolished (complete entrance block) (*Figure 2D*). Pacing from the CMC at an output of 10 V inside the SVC isolation line did not establish conduction to the atrium (complete exit block) (*Figure 2E*). Programmed stimulation with bolus injections of isoproterenol no longer induced AF. The patient remained well without AF recurrence for >9 months after ablation under oral administration of bisoprolol (2.5 mg), pitavastatin (2 mg) once daily, and apixaban (5 mg) two times daily.

## Discussion

The SVC has myocardial sleeves that extend 40–50 mm cranially into the vein.^2^ However, the SVC myocardium has a different origin than the myocardial sleeves of the PVs; hence, the arrhythmogenic potential of the SVC is not as prominent as that of the PVs.^2^ The SVC acts as a non-PV trigger of AF in 2%–6% of patients with AF and harbours 25%–40% of non-PV foci, which are associated with AF recurrence.^2,6^ The patient had no AF recurrence for 3 years after the first session and had several cardiovascular risk factors, including a history of cerebral infarction, hyperlipidaemia, and older age. These conditions may cause the progression of electrical and structural remodelling of the SVC, which may accelerate the action of non-PV triggers in AF. Further, the increased length of SVC (≥37 mm) myocardial sleeves and increased SVC diameter (≥17 mm) are reported as independent predictors of SVC firing in patients with AF.^6^ In the current case, these were 41 and 18 mm, respectively, indicating a high risk of non-PV triggers of AF.^2,6^

Moreover, recent reports have demonstrated that ∼10% of patients with AF have ECs between the atrium and PV inside the PVAI lines, which play an important role in initiating and maintaining AF.^5,7^ However, the EC(s) between the SVC and RA that contribute to subsequent AF remain unreported. Therefore, distinguishing between ECs inside the SVC isolation lines and gap conduction along the SVC isolation lines after SVC isolation is crucial. In the current case, the location of the EC in the SVC was >5 mm (18 mm) from the SVC isolation line.^5,7,8^ Additionally, local activation time isochronal mapping during pacing using an ablation catheter on the EC in the SVC demonstrated that the earliest activation site was the RA (*Figure 2C*) without electrical endocardial conduction (*Figure 1B*). Finally, after EC ablation of the SVC, we confirmed the complete entrance (*Figure 2D*) and exit (*Figure 2E*) blocks of the SVC. Thus, gap conduction between the SVC and RA is unlikely. Moreover, a recent report has demonstrated that distinguishing between ECs inside the PVAI lines and non-transmural lesions along the PVAI lines after PVAI is crucial. The LA wall thickness varies from 1.3 to 6.5 mm.^7,9^ Consequently, the definition of ECs may encompass true ECs pre-existing prior to PVAI in addition to false ECs formed after PVAI as residual epicardial-sided conduction related to non-transmural lesion creation because of a thicker LA wall thickness.^7^ In the present case, the SVC wall measured using preprocedural CT was thin (1.3–1.5 mm). Thus, a non-transmural lesion of the SVC is unlikely.

Although the mechanisms of ECs in the SVC and RA have not been entirely elucidated, several potential mechanisms have been proposed. The ECs, such as the Bachmann bundle, septopulmonary bundle, Marshal bundle, or intercaval bundle, connect the PV(s) and atrium on the epicardial side.^2,7^ Thus, anatomically inherited ECs may run through the epicardial side between the SVC and RA. These ECs are anatomically inherited and predominantly situated near the right- and left-sided PV carina.^5,7,8^ The ECs between right PV and SVC^10^ or RA^5,7^ have been recently reported. In the second session, we confirmed the complete entrance and exit blocks of the four PVs, indicating complete ablation of the right PV carina by CBA. Thus, we might speculate on the possibility of the existence of EC(s) between the right-sided PV carina and both the SVC and RA. After the complete ablation of the right-sided PV carina in the first session, the connection between the SVC and RA remained and acted as an EC. Further investigation is required to clarify the poorly understood EC(s) between the SVC and RA.

Lastly, the recent advancements in the new technologies including pulsed-field ablation can facilitate a more stable and deeper lesion creation with non-transmural lesion creation.^11^ Thus, pulsed-field ablation-based SVC isolation might be a feasible and safe alternative strategy to isolate SVC, since it reduces the risk of the phrenic nerve palsy and sinus node dysfunction.^12^

## Conclusion

Physicians should consider the possibility of EC(s) when remaining potentials in the SVC are observed, even though the SVC isolation line seems to be completed.

## Lead author biography


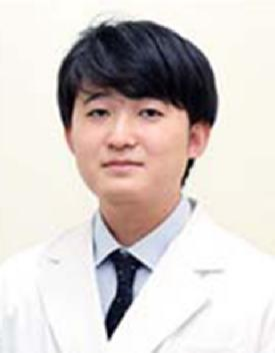


Dr Shunya Otsubo received his MD degree from Kyushu University (Fukuoka, Japan) in 2022. Since 2024, he has been working as a cardiologist at the Social Medical Corporation Steel Memorial Yawata Hospital. His area of medical interest is arrhythmias, heart failure, and coronary artery disease.

## Data Availability

The deidentified participant data will not be shared.
